# Attitude, Beliefs, and Use of Herbal Remedies by Patients in the Riyadh Region of Saudi Arabia

**DOI:** 10.3390/healthcare10050907

**Published:** 2022-05-13

**Authors:** Ahmad H. Alghadir, Amir Iqbal, Zaheen A. Iqbal

**Affiliations:** Department of Rehabilitation Sciences, College of Applied Medical Sciences, King Saud University, P.O. Box 10219, Riyadh 11433, Saudi Arabia; alghadir@ksu.edu.sa (A.H.A.); z_iqbal001@yahoo.com (Z.A.I.)

**Keywords:** patients, herbal medicines, attitude, belief, side effects

## Abstract

Background: The consumption of herbal medicines (HMs) is increasing worldwide, especially in developing countries. This study attempts to investigate and evaluate the patient’s perception with regard to the security of HMs, their attitudes towards the concomitant use of HMs with modern medicines, and counseling about their use. Design: Self-administered questionnaire-based cross-sectional survey study. Setting: A self-administered structured questionnaire was administered to 200 patients who received HMs from four different government and private hospitals in the Riyadh region of Saudi Arabia, over a period of three months. Results: The response rate was 74.5%. Out of these, 76.83% of respondents reported using HMs in some form for a variety of conditions. There was no statistically significant relationship between various demographic characteristics and the use of herbs. The majority of the respondents (76.72%) reported using HMs without any professional supervision. This exposes them to the risk of harmful side effects and drug interactions. Conclusions: Physicians and pharmacists should work to provide evidence-based information about HMs to patients about effectiveness and side effects and be vigilant while writing prescriptions and dispensing drugs to them. Patient counseling and education about medication use are required to augment their awareness about their use.

## 1. Introduction

The use of herbal medicines (HMs) continues to expand globally with their increased acceptance among consumers [1]. Although HMs are not classified as drugs by the US Food and Drug Administration (FDA), they are considered to be safe for use among the general population as they are natural products, derived from nature [2,3,4]. It has been reported that 30–50% of the population in developed countries use HMs [5], and according to various reports, nearly USD 4 billion are spent on purchasing these products every year [6].

The healthcare system in Saudi Arabia (SA), as in most Middle Eastern countries, is primarily based on a modern medicine system; nonetheless, traditional, locally made HMs are very popular among locals [7,8]. Products that make medicinal claims or contain active ingredients that may exhibit medicinal effects, such as herbal preparations, health and food supplements, medicated cosmetics, antiseptics and medical devices, are required to be registered with the Ministry of Health (MOH) [7,8]. In contrast to modern medicines, clinical practitioners generally do not have much scientific data regarding the risks and benefits of HMs as they do not undergo rigorous clinical trials and post-approval surveillance to determine their effectiveness and relative safety [6,9]. Hence, the safety of HMs has now become a major concern among health-regulatory bodies, professionals and consumers.

Riyadh is the capital city, located in the central region of Saudi Arabia, with a population of 7.39 million, corresponding to 24.9% and 84.36% of the population of the country and its central region, respectively [10]. The capital’s population is mixed, coming from all corners of Saudi Arabia, representing a vast range of cultural attitudes, customarily beliefs, traditional practices and different lifestyles depending on the traditional culture, occupations and education [10].

Although several studies report public interest towards HMs, the attitudes and perceptions of the patients towards these products have not been adequately addressed in the region [1,2,3,4,5,6,7,8,9]. To the best of our knowledge, no studies have been designed to evaluate the current level of knowledge and awareness about HMs among consumers in Saudi Arabia and their attitudes and beliefs towards combining them with modern medicines. Therefore, objective of this study was to investigate the patient’s perception with regard to the security of HMs. In addition, we also evaluated the attitudes towards the concomitant use of HMs with modern medicines, and the counseling about their use.

## 2. Materials and Methods

### 2.1. Participants

**Scheme 1:**
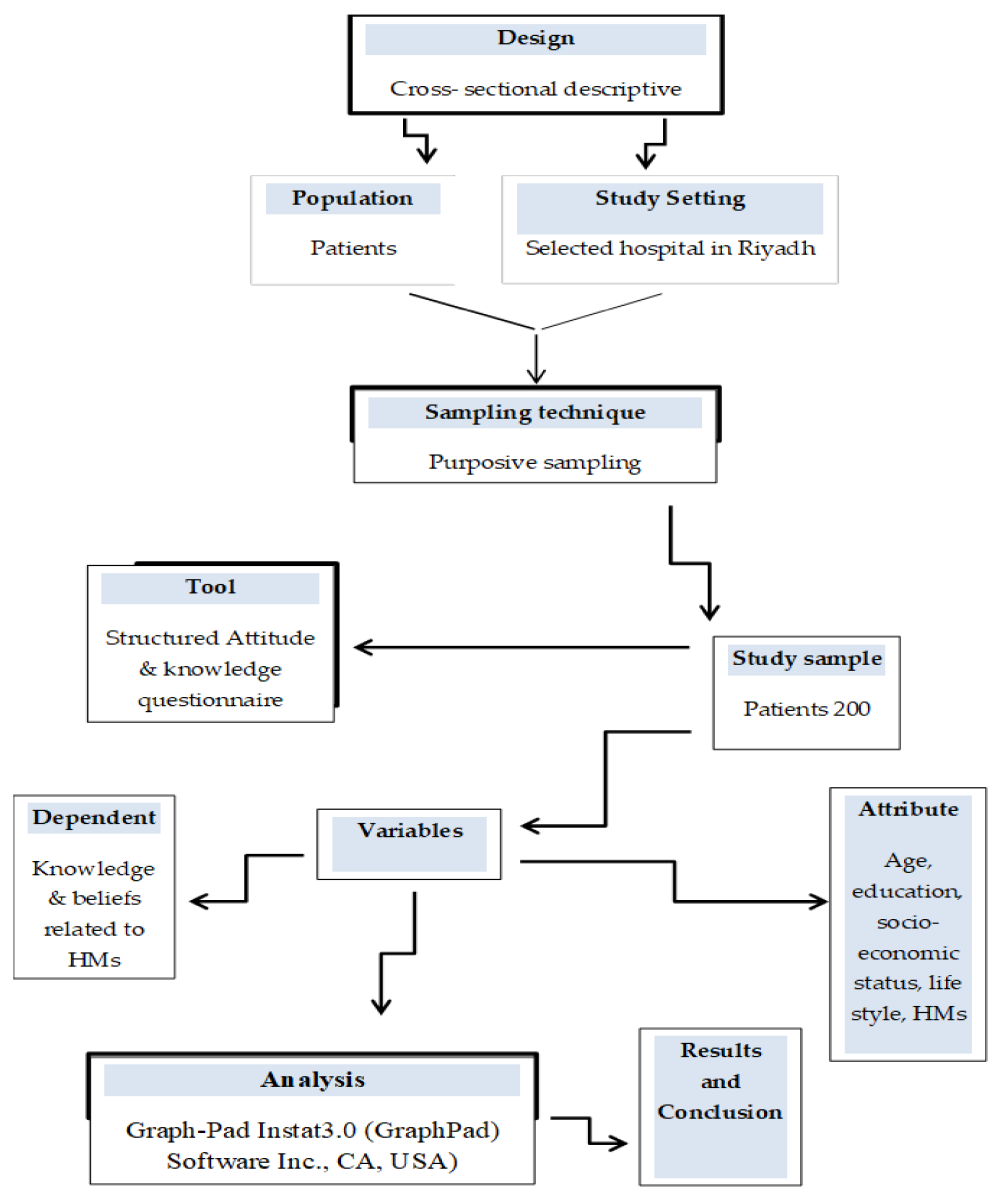
An outline of the research design.

Two hundred patients who were receiving HMs by attending an outpatient clinic, hospital or follow-up in four government and private hospitals in the central region (Riyadh), Saudi Arabia, over a period of three months from April 2014 were invited to participate in the study. These hospitals were randomly selected for visits according to their location. An outline of the research design is shown in Scheme 1. The purpose and aim of the study were explained to the participants and their written informed consent was obtained. The study was conducted per the Declaration of Helsinki (2010) and in line with the rights and ethics for conducting human research accordingly. It was approved by the Ethics Sub-Committee of King Saud University (RRC-2014-008; dated 11 March 2014).

### 2.2. Study Tool

Adult participants aged between 18 and 65 years, of both sexes (males and females), and who showed voluntary cooperation, were included in this study. Meanwhile, participants aged less than 18 years and more than 65 years and who showed non-cooperation were excluded from this study. A self-administered structured questionnaire (Arabic version) was used to evaluate the knowledge, attitudes and beliefs of participants towards the sources, use, safety and side effects of the herbal medicines was used. It was a reliable (Cronbach’s α coefficient 0.871) tool adopted from similar studies conducted among the population of Arabian countries and other parts of the world [7,8,9,11,12]. It consisted of 4 sections: demographic data, attitudes of the respondents towards the sources, safety and their need to consult a physician or pharmacist about HMs, use of HMs and their beliefs about their side effects. The language of the questionnaire was Arabic, and it consisted of various closed-ended and multiple-choice questions. The term HMs was used throughout the survey to exclude any vitamin and mineral use. The questionnaire was translated from English to the Arabic language first, followed by the English language again to preserve the actual meaning of the questionnaire for collecting data. A panel of senior researchers at the pharmacy department of King Saud University reviewed it for its appropriateness, accuracy and efficiency for collecting accurate data among the Arab population. A pilot study was conducted among 23 patients to confirm that it fulfilled the requirements of the study and minor changes were made depending on the results. Further, in order to assess the reliability of the questionnaire tool, the internal consistency was found to be good as the Cronbach’s α coefficient was 0.839. The data of the respondents involved in the pilot study were not included in the assessment/analysis of this study.

### 2.3. Statistical Analysis

Data were analyzed using GraphPad Instat 3.0 (GraphPad Software Inc., La Jolla, CA, USA). Means, frequencies and percentages were used for descriptive statistics. The chi-square (χ^2^) test was performed to determine significant statistical differences between groups of respondents. *p* value < 0.05 indicated the level of significance.

## 3. Results

### 3.1. Demographic Characteristics and Use of HMs Based on Age and Gender of the Respondents

Out of 200 patients who were approached for the study, a total of 149 participants (with a response rate of 74.5%) voluntarily agreed to participate. More than half of the respondents were males (55.03%) and greater than 40 years of age (38.26%). Similarly, more than half of respondents were married (62.42%), compared with single (18.12%), divorced (10.74%) and widowers (8.72%); 43.62% belonged to the annual income group of SAR 3000.00–6000.00, compared with SAR < 3000.00 (34.9%) and SAR > 6000.00 (21.48%). The majority of respondents were educated and had a bachelor degree (45.64%) compared with unlettered (6.04%), primary school (10.07%), secondary school (28.19%) and postgraduates (10.07%). Moreover, 54.36% of the respondents reported to be visiting outpatient clinics compared with departments in hospitals (20.81%) and follow-ups (24.83%). Despite representing a lesser percentage than males, the female respondents (79.10%) were reported as more frequent users of HMs. Noticeably, the age group of more than 40 years (82.46%) were reported as more frequent users of HMs compared to other age groups. Further, more details on the demographic characteristics and the rate of uses and non-uses of HMs according to gender and age are shown in Table 1.

### 3.2. Age-Wise Distribution of Gender and Education Level of the Respondents

More females (34.33%) than male respondents were reported in the age group below 30 years. However, more males (45.12%) than female respondents were registered in the age group above 40 years. Interestingly, fewer female (7%) and male (12%) respondents were reported in the 36–40 years age group. The highest percentage of respondents (27%) were graduates, reported as in the age group above 40 years. However, the lowest percentage of respondents (0.00%) were unlettered, reported in the age group below 30 years. Furthermore, Table 2 details the age-wise distribution of the gender and education level of the respondents.

### 3.3. Patients’ Attitudes towards HMs

In response to the question on the use of HMs, 116 (77.85%) participants reported to have used them before in some form, 50% of which reported to have used them for more than 2 months at a time continuously, as shown in Table 3. The majority of the patients decided to use HMs based on their own research on the internet (31.90%), family advice (19.83%), doctors (16.38%) or by recommendations from friends (15.52%). Among the total respondents, 48.28% reported that they discussed the use and effects of HMs with a pharmacist or physician and at least 53.44% reported to be somewhat satisfied with their use. In addition, 83.62% and 85.34% of respondents also reported that they did not have any difficulty in obtaining HMs in the market and that it was appropriate and affordable, respectively. Results about respondents’ knowledge regarding the origin and adulteration of HMs were somewhat surprising, with at least 28.45% of them reporting that they did not care about these aspects. However, 26.72% and 8.62% of respondents reported to depend on pharmacists and doctors, respectively, for knowledge about such details of HMs.

### 3.4. Use of HMs

More than half of the respondents (57.75%) reported that they never used HMs as a dietary supplement. The most common conditions for which respondents reported the use of HMs included hypertension (18.10%), diabetes (15.52%) and urinary tract infections (15.52%), followed by other conditions including migraine (11.21%), skin diseases (10.34), loss of appetite (9.48), metabolism (6.90%) and reproductive (9.48) and nervous system problems (3.45%). At least 76.72% of respondents indicated that they did not consult their physicians before using HMs. Around half of the respondents (52.59%) reported that they used HMs when necessary, while 20.69% reported that they used them regularly. The majority of respondents (68.96) used HMs once on a daily (68.96%) and weekly (6.55%) basis, most commonly in the form of capsules (37.07%). Furthermore, all responses related to the use of HMs among the respondents are presented in Table 4.

### 3.5. Patients’ Beliefs on Side Effects of HMs

Our questionnaire included four questions regarding the beliefs of patients towards the side effects of HMs. Around 31.9% of the respondents reported that they found improvements in their health to a certain extent, while 13.79% reported that it worsened their condition. In spite of reporting no improvement in health, 42.24% of respondents reported that they restarted the HMs again. At least 67.24% of the respondents were reported to believe that the concurrent use of HMs and modern medicines is safe and can help to speed up recovery, while 19.83% reported that the use of HMs alone had improved their health condition. Further, details regarding the side effects of HMs reported by respondents included headache (20.70%), dizziness (18.97%) and urine discoloration (14.66%), etc., as described in Table 5.

## 4. Discussion

Throughout Saudi Arabia, HMs are freely available to the general public through pharmacies and other shops in retail markets. However, MOH can only control their dispensing at registered pharmacies. Hence, a substantial number of unregistered HMs are available to patients for a variety of health conditions. The popularity of HMs for the treatment of patients with chronic diseases may be attributed to the long-standing suffering of the patients or failure of medical treatment to bring about quick and long-lasting relief [11,12].

Our study aimed to supply descriptive data on the status of the attitudes, beliefs and knowledge of patients in four renowned hospitals from every area in the Riyadh region of Saudi Arabia. Our results show that respondents older than 40 were more educated than other age groups who used HMs more frequently. This has been shown in other studies as well, which emphasizes the need for improved knowledge of practitioners about HMs [13,14]. This can be achieved through appropriate educational activities about HMs for patients as well as healthcare professionals [15]. A similar study conducted in the region—the Siruvani Hills of the Western Ghats of India—regarding the use of certain medicinal plants revealed a significant relationship between the use of medicinal plants and education among tribal people compared to non-tribal people [16]. It also emphasized that, since tribal people have lifelong familiarity with medicinal plants, they have more knowledge about them, so they show different attitudes and more confidence in the use of HMs.

The use of HMs is popular in the central region of Saudi Arabia, with a widespread belief that HMs are natural and therefore safe. Results further show that at least 77.85% of the respondents had considered the use of HMs sometime in their lifetime and 42.24% used them as dietary supplements. The word ‘natural’ is commonly associated with purity, safety and the absence of harmful chemicals or preservatives [1,4,9]. Another study [17] in Saudi Arabia also reported that at least 24% of the patients attending health centers had used some local alternative remedy. However, no statistically significant relationship could be established between the use of HMs and patient demographic characteristics such as gender, age, location of residence and education level. Similarly, there was no significant difference between the HM user and HM non-user respondents. However, a previous study conducted in Kashan city of Iran, on the use of HMs among diabetic type 2 patients, reported a significant association between the use of HMs and education level and place of residence [12] (Table 1 and Table 2).

Anecdotally, the increased prevalence of self-medication has been reported in Saudi Arabia [3,18,19,20]. In a community-based survey in the central region of Saudi Arabia, one study reported that almost 85–91% respondents claimed to be users of HMs without any consultation with physician or pharmacists [13,17]. Similarly, in our study, only 76.72% of the respondents reported that they did not discuss the use of HMs with their physicians. This promotes the concomitant use of HMs and modern medicines, raising the chances of potential, harmful interactions of drugs, which may create pharmacological and toxicological effect on each other [11,20,21]. A great number of interactions between such medicines have been confirmed [22]. Patients generally use HMs to augment the effects of modern medicines, rather than due to their dissatisfaction about their use [23,24]. However, a recently published systematic review study pointed out the dissatisfaction with conventional medicines and positive attitudes towards complementary alternative medicines (CAM) as the reason for using CAM worldwide [12]. Moreover, many countries, such as the USA, Kenya, Japan, etc., have been raising concerns over the increasing prevalence and cost of using alternative medicines including HMs based on their self-medication [25,26,27,28].

Our results also show that a total of 72.42% respondents decided to use HMs based on their own research on the internet or due to the advice of family and friends, which is supported by previous studies conducted in Saudi Arabia [18,21]. The increased availability and aggressive advertising campaigns of such preparations also contribute to the higher number of consumers [29], without proper consultations with health practitioners [14,18]. It has been reported that patients are afraid to discuss the concomitant use of different medicines out of fear of their disapproval [22,30,31]. This also affects the regularity and duration of their use, with 20.69% of respondents reported to use them regularly and 50% for more than two months, which cannot be controlled. We propose that HMs should only be available at pharmacies upon presentation of a valid prescription, as with other modern medicines.

The chemical composition of HMs varies with their preparations. It depends on various biological factors, such as its origin, the part of the plant used or the time of harvest [30]. HMs have been described as complex pharmaceutical products in the literature [32]. Respondents also reported that they least cared about the purity of available HMs before their use. Besides this, an increased number of cases involving various side effects of such drugs have been reported [33].

Respondents have expressed their desire that HMs should be easy to obtain and should be available at a cheaper price [11]. Another study among general practitioners has also revealed that the wide usage and acceptance of HMs may be further promoted by their low cost and evidence of their effectiveness [11,12]. The availability of HMs in the form of tablets, capsules and extracts, in contrast to initial crude forms, has also contributed to their increased use among consumers. HMs in the form of capsules and powder have been reported to be more popular among respondents.

The prevalence of chronic illnesses such as diabetes and cardiovascular diseases is on the rise in Saudi Arabia [34,35], due to lifestyle changes in recent times. This leads to a rise in the number of patients using multiple medications 2. Therefore, more positive attitudes from physicians towards HMs will not only promote research regarding the effectiveness of HMs in various diseases, but also encourage patients to talk to them about their concomitant use [11,36]. This shall help in preventing various harmful drug interactions, which can be hazardous for patients [11,37,38,39,40].

Despite being a proper descriptive cross-sectional survey study design, this study was limited to the patients taken from various renowned hospitals located in the Riyadh region—the capital city of Saudi Arabia. Therefore, this study cannot be generalized to the whole population of Saudi Arabia. Additionally, this study was limited to observing the attitudes, beliefs and practices of health practitioners in recommending the use of HMs. Therefore, future research based on a cross-sectional study design must include optimal numbers of patients from all regions of Saudi Arabia to obtain more concrete, accurate and up-to-date results and information regarding (1) the patient’s attitudes, beliefs and use of HMs, and (2) the health practitioner’s attitudes and practices in recommending HMs.

## 5. Conclusions

Although respondents report the use of HMs for a variety of conditions, most of the time, they are used without any professional supervision. This exposes them to the risk of harmful side effects and drug interactions if used concomitantly with modern medicines. Doctors and pharmacists should work to provide evidence-based information about HMs to patients about their effectiveness and side effects and be vigilant while writing prescriptions and dispensing drugs to them. Patient counseling and education about medication use is required to augment their awareness about their use. This study needs to be repeated in other areas of Saudi Arabia with a larger number of respondents belonging to different categories.

## Figures and Tables

**Table 1:** Demographic characteristics and use of HMs based on age and gender of the respondents, using chi-square (χ^2^) tests (*N* = 149).

Sl.No.	Demographic Characteristics (*N* = 149)		Gender			Age (Years)				
			Male	Female	*p*-Value	<30	30–35	36–40	>40	*p*-Value
1.	Number (%)		82 (55.03)	67 (44.97)	0.896	38 (25.5)	35 (23.48)	19 (12.75)	57 (38.26)	0.627
2.	Use of HMs, *N* (%)	Yes	63 (76.83)	53 (79.10)		27 (71.05)	27 (77.14)	15 (78.95)	47 (82.46)	
		No	19 (23.17)	14 (20.90)		11 (28.95)	8 (22.86)	4 (21.05)	10 (17.54)	

Not significant if *p* > 0.05, *N*: Number of participants.

**Table 2:** Age-wise distribution of gender and education level of the respondents (*N* = 149).

Age	Gender, *N* (%)		Education, *N* (%)				
	Females	Males	Unlettered	Primary School	Secondary School	Graduation	Post-Graduation
<30	23 (34.33)	15 (18.29)	0(0)	9 (60.00)	15 (35.71)	12 (17.65)	2 (13.33)
30–35	17 (25.37)	18 (21.95)	3 (33.33)	1 (6.67)	11 (26.19)	18 (26.47)	2 (13.33)
36–40	7 (10.45)	12 (14.63)	3 (33.33)	1 (6.67)	1 (2.38)	11 (16.18)	3 (20.00)
>40	20 (29.85)	37 (45.12)	3 (33.33)	4 (26.66)	15 (35.71)	27 (39.70)	8 (53.34)
Total (*N*)	67(44.97)	82(55.03)	9 (6.04)	15 (10.07)	42 (28.19)	68 (45.64)	15 (10.07)

*N*: Number of participants.

**Table 3:** Attitudes of respondents towards HMs and their information sources (*n* = 149).

Sl. No.	Questions Asked	Number (%)						
1.	Have you used medicine before?	No 33 (22.15)			Yes 116 (77.85)			
2.	If yes, how long?	1 week 14 (12.06)	2 weeks 17 (14.66)	1 month 8 (6.90)	2 months 19 (16.38)		>2 months 58 (50.0)	
3.	Sources of your information about HMs?	Doctors 19 (16.38)	Internet 37 (31.90)	Friends 18 (15.52)	Family 23 (19.83)	Myself 6 (5.17)	Pharmacist 10 (8.62)	Center 9 (7.76)
4.	How do you know about Original or fake HMs?	Doctors 10 (8.62)	Insuring brand 5 (4.31)	Day of manufacture 14 (12.06)	Form 11 (9.48)	smell 14 (12.06)	Pharmacist 31 (26.72)	I don’t care 33 (28.45)
5.	The price of HMs’	Appropriate 99 (85.34)			Expensive 17 (14.66)			
6.	Did you find difficult to obtain HMs?	No 97 (83.62)			Yes 19 (16.38)			
7.	How many times, did you discuss with doctor or Pharmacist?	None 28 (24.14)	1 time 11 (9.48)		3 times 21 (18.10)		More than one time 56 (48.28)	
8.	Are you satisfied with the HMs prescribed?	Totally satisfied 27 (23.28)		Somewhat satisfied 62 (53.44)			Dissatisfied 27 (23.28)	

**Table 4:** Use of HMs by respondents (*n* = 116 out of 149).

Sl. No.	Questions Asked		Responses, *n* (%)			
1.	Do you use the HMs as dietary Supplement?		Yes 49 (42.24)		No 67 (57.75)	
2.	Do you confirm with your physician after purchasing the HMs?		Yes 27 (23.28)		No 89 (76.72)	
3.	How often you use the HMs?		Regularly 24 (20.69)	If necessary 61 (52.59)	Irregularly 31 (26.72)	
4.	Intake of HMs.	Daily	Once 80 (68.96)	Twice 27 (23.28)	Trice 9 (7.76)	Others 0 (0)
		Weekly	Once 54 (46.55)	Twice 25 (21.55)	Trice 37 (31.90)	Others 0 (0)
5.	You took your HMs as		Capsules 43 (37.07)	Bags 35 (30.17)	Powder 38 (32.76)	Others 0 (0)

HMs: herbal medicines.

**Table 5:** Respondents’ beliefs about side effects of HMs (*n* = 116 out of 149).

Sl. No.	Questions Asked	Response, *n* (%)				
1.	After taking HMs my health condition	Improved strongly 32 (27.58)	Improved 18 (15.52)	Improved to a certain extent 37 (31.90)	Did not improve 13 (11.21)	Worsened health 16 (13.79)
2.	My health condition improved with	Conventional drugs 15 (12.93)		Herbal medicines 23 (19.83)		Both together 78 (67.24)
3.	What did you do when worsening your health condition	Stopped talking HMs by myself 32 (27.59)		Revised the doctor 35 (30.17)		Used again 49 (42.24)
4.	Side effects caused to me when using HMs	Diarrhea 7 (6.03)	Constipation 1 (0.86)	Vomiting 1 (0.86)	Headache 24 (20.70)	
		Dizziness 22 (18.97)	Blurring of eyes 9 (7.76)	Change in mood 11 (9.48)	Prickle 6 (5.17)	
		Skin redness 1 (0.86)	Muscular pain 1 (0.86)	Nose bleeding 1 (0.86)	Heart palpitation 2 (1.72)	
		Increased appetite 5 (4.31)	Overweight 3 (2.59)	Increased urination 5 (4.31)	Change in urine color 17 (14.66)	

HMs: herbal medicines.

## Data Availability

The data presented in this study are available on request from the corresponding author. The data are not publicly available due to privacy reason.

## References

[1] Pal S.K., Shukla Y. (2003). Herbal medicine: Current status and the future. Asian Pac. J. Cancer Prev..

[2] Barnes P.M., Powell-Griner E., McFann K., Nahin R.L. (2004). Complementary and alternative medicine use among adults: United States, 2002. Seminars in Integrative Medicine.

[3] Al-Arifi M.N. (2013). Availability and needs of herbal medicinal information resources at community pharmacy, Riyadh region, Saudi Arabia. Saudi Pharm. J. Off. Publ. Saudi Pharm. Soc..

[4] Rivera J.O., Loya A.M., Ceballos R. (2013). Use of herbal medicines and implications for conventional drug therapy medical sciences. Altern. Integr. Med..

[5] Robinson M.M., Zhang X. (2011). The World Medicine Situation 2011 Traditional Medicine: Global Situation, Issues and Challenges.

[6] Barnes P.M., Bloom B., Nahin R.L. (2008). Complementary and alternative medicine use among adults and children: United States, 2007. Natl. Health Stat. Rep..

[7] Alghamdi M., Mohammed A.A., Alfahaid F., Albshabshe A. (2018). Herbal medicine use by Saudi patients with chronic diseases: A cross-sectional study (experience from Southern Region of Saudi Arabia). J. Health Spec..

[8] Ullah R., Alqahtani A.S., Noman O.M., Alqahtani A.M., Ibenmoussa S., Bourhia M. (2020). A review on ethno-medicinal plants used in traditional medicine in the Kingdom of Saudi Arabia. Saudi J. Biol. Sci..

[9] Alkhamaiseh S.I., Aljofan M. (2020). Prevalence of use and reported side effects of herbal medicine among adults in Saudi Arabia. Complementary Ther. Med..

[10] Riyadh Population 2022 (Demographics, Maps, Graphs)—World Population Review. https://worldpopulationreview.com.

[11] Hill J., Mills C., Li Q., Smith J.S. (2019). Prevalence of traditional, complementary, and alternative medicine use by cancer patients in low income and lower-middle income countries. Glob. Public Health.

[12] Tangkiatkumjai M., Boardman H., Walker D.-M. (2020). Potential factors that influence usage of complementary and alternative medicine worldwide: A systematic review. BMC Complementary Med. Ther..

[13] Picking D., Younger N., Mitchell S., Delgoda R. (2011). The prevalence of herbal medicine home use and concomitant use with pharmaceutical medicines in Jamaica. J. Ethnopharmacol..

[14] Suleiman A. (2014). Attitudes and beliefs of consumers of herbal medicines in Riyadh, Saudi Arabia. J. Community Med. Health Educ..

[15] Shields K.M., McQueen C.E., Bryant P.J. (2004). National survey of dietary supplement resources at drug information centers. J. Am. Pharm. Assoc..

[16] Navaraj P. (2003). Attitudes towards the Use of Medicinal Plants for Diseases in the Siruvani Hills of Western Ghats, India.

[17] Al-Ajaji N., Taha A.Z., Al-Zubier A.G. (1998). Prevalence of utilization of native medicine among primary care consumers. Saudi Med. J..

[18] Al-Yousef H.M., Wajid S., Sales I. (2019). Knowledge, Beliefs and Attitudes towards Herbal Medicine—A Community-based Survey from a Central Region of Saudi Arabia. Indian J. Pharm. Pract..

[19] Thomson P., Jones J., Evans J.M., Leslie S.L. (2012). Factors influencing the use of complementary and alternative medicine and whether patients inform their primary care physician. Complementary Ther. Med..

[20] Welz A.N., Emberger-Klein A., Menrad K. (2018). Why people use herbal medicine: Insights from a focus-group study in Germany. BMC Complementary Altern. Med..

[21] Al Akeel M.M., Al Ghamdi W.M., Al Habib S., Koshm M., Al Otaibi F. (2018). Herbal medicines: Saudi population knowledge, attitude, and practice at a glance. J. Fam. Med. Prim. Care.

[22] Yilmaz M.B., Yontar O.C., Turgut O.O., Yilmaz A., Yalta K., Gul M., Tandogan I. (2007). Herbals in cardiovascular practice: Are physicians neglecting anything?. Int. J. Cardiol..

[23] Astin J.A. (1998). Why patients use alternative medicine: Results of a national study. JAMA.

[24] Druss B.G., Rosenheck R.A. (1999). Association between use of unconventional therapies and conventional medical services. JAMA.

[25] Kigen G.K., Ronoh H.K., Kipkore W.K., Rotich J.K. (2013). Current trends of traditional herbal medicine practice in Kenya: A review. Afr. J. Pharmacol. Ther..

[26] Rashrash M., Schommer J.C., Brown L.M. (2017). Prevalence and predictors of herbal medicine use among adults in the United States. J. Patient Exp..

[27] Gunjan M., Naing T.W., Saini R.S., Ahmad A., Naidu J.R., Kumar I. (2015). Marketing trends & future prospects of herbal medicine in the treatment of various disease. World J. Pharm. Res..

[28] Khan M.S.A., Ahmad I. (2019). Herbal medicine: Current trends and future prospects. New Look to Phytomedicine.

[29] Williamson E.M., Rankin-Box D. (2009). Complementary therapies, the placebo effect and the pharmacist. Complementary Ther. Clin. Pract..

[30] Bozin B., Mimica-Dukic N., Bogavac M., Suvajdzic L., Simin N., Samojlik I., Couladis M. (2008). Chemical composition, antioxidant and antibacterial properties of Achillea collina Becker ex Heimerl s.l. and A. pannonica Scheele essential oils. Molecules.

[31] Eisenberg D.M., Kessler R.C., Foster C., Norlock F.E., Calkins D.R., Delbanco T.L. (1993). Unconventional medicine in the United States. Prevalence, costs, and patterns of use. N. Engl. J. Med..

[32] Samojlik I., Mijatovic V., Gavaric N., Krstin S., Bozin B. (2013). Consumers’ attitude towards the use and safety of herbal medicines and herbal dietary supplements in Serbia. Int. J. Clin. Pharm..

[33] Edwards R. (1995). Monitoring the safety of herbal remedies. WHO project is under way. BMJ.

[34] Al-Nozha M.M., Abdullah M., Arafah M.R., Khalil M.Z., Khan N.B., Al-Mazrou Y.Y., Al-Maatouq M.A., Al-Marzouki K., Al-Khandra A., Nouh M.S. (2007). Hypertension in Saudi Arabia. Saudi Med. J..

[35] Al-Nozha M.M., Al-Maatouq M.A., Al-Mazrou Y.Y., Al-Harthi S.S. (2004). Diabetes mellitus in Saudi Arabia. Saudi Med. J..

[36] Azizi-Fini I., Adib-Hajbaghery M., Gharehboghlou Z. (2016). Herbal medicine use among patients with type 2 diabetes in Kashan, Iran, 2015. Eur. J. Integr. Med..

[37] Izzo A.A., Di Carlo G., Borrelli F., Ernst E. (2005). Cardiovascular pharmacotherapy and herbal medicines: The risk of drug interaction. Int. J. Cardiol..

[38] Izzo A.A., Ernst E. (2009). Interactions between herbal medicines and prescribed drugs: An updated systematic review. Drugs.

[39] Zhou S., Gao Y., Jiang W., Huang M., Xu A., Paxton J.W. (2003). Interactions of herbs with cytochrome P450. Drug Metab. Rev..

[40] Spolarich A.E., Andrews L. (2007). An examination of the bleeding complications associated with herbal supplements, antiplatelet and anticoagulant medications. J. Dent. Hyg..

